# Structures of a highly variable cell‐wall anchored protein‐encoding the *spj* gene from ST8/SCC*mec*IVl community‐associated methicillin‐resistant *Staphylococcus aureus* (CA‐MRSA/J) isolated from 2003 onwards: An indicator of a strongly invasive pathotype

**DOI:** 10.1111/1348-0421.12684

**Published:** 2019-05-23

**Authors:** Tsai‐Wen Wan, Lee‐Jene Teng, Tatsuo Yamamoto

**Affiliations:** ^1^ Department of Epidemiology Genomics, and Evolution, International Medical Education and Research Center Niigata Japan; ^2^ Laboratory Sciences and Medical Biotechnology National Taiwan University College of Medicine Taipei Taiwan

**Keywords:** cell wall‐anchored protein, community‐associated methicillin‐resistant *Staphylococcus aureus*, pUB110, staphylococcal cassette chromosome *mec* IVl

## Abstract

The cell wall‐anchored protein‐encoding *spj* gene on staphylococcal cassette chromosome *mec* IVl (SCC*mec*IVl) was found to vary in size because of its 22‐ and 86‐aa repeat domains. The 22‐aa repeats are the more flexible of the two repeats, comprising three 11‐aa units, and were classified into three groups with eleven types. The 11/22‐aa repeats are longer in individuals with bullous impetigo, shorter in those with invasive disease and were absent in a fatal case, this last one having been rapidly diagnosed by PCR. IS*431*‐flanking pUB110 (*bleO*, *aadD*) is present on SCC*mec*IVl at 90%. The bacterial surface has the *spj* product and a unique surface layer.

**Abbreviations**
BSI: blood stream infection
CA‐MRSA: community‐associated methicillin‐resistant *Staphylococcus aureus*
CWA protein: cell wall‐anchored protein
HA‐MRSA: healthcare‐associated methicillin‐resistant *Staphylococcus aureus*
J1 region: joining region 1 (of SCCmec)
SCCmec IVl: staphylococcal cassette chromosome *mec* IV subtype l
SEM: scanning electron microscopy
SSTI: skin and soft tissue infection
TEM: transmission electron microscopy

The term MRSA includes healthcare‐ and community‐associated types, HA‐ and CA‐MRSA, respectively. HA‐MRSA infections most frequently occur in inpatients,^1^ whereas CA‐MRSA infections occur in healthy individuals, usually causing SSTIs, but occasionally causing invasive infections.^1^, ^2^ HA‐ and CA‐MRSA carry the SCC*mec*.^3^ SCC*mec*IV is common among CA‐MRSA, in contrast to SCC*mec*I to III.^2^ CA‐MRSA, particularly USA300 with ST8/SCC*mec*IVa, was responsible for serious disease outbreaks in the USA in 2007.^1^, ^2^, ^4^ Currently, the role of SCC*mec*IV in CA‐MRSA pathogenesis remains unclear.

We previously reported on ST8/SCC*mec*IVl CA‐MRSA (CA‐MRSA/J) in 2012.^5^, ^6^ CA‐MRSA/J is associated with a broad range of disease manifestations,^6^, ^7^ one death having been caused by a strongly invasive pathotype,^8^ and can be transmitted on public transport,^9^ including having been spread internationally to Hong Kong.^6^ A key feature of SCC*mec*IVl is a large CWA protein encoding the *spj* gene.^5^, ^6^ Although we analyzed the genome of the invasive NN50 strain, isolated in 2008,^6^ initial strains (NN3 and NN4) isolated from bullous impetigo in 2003 10 and SI1 strain from the first fatal case in 2012 8 await investigation. In the present study, we analyzed the SCC*mec*IVl structures of these four strains, together with 26 other CA‐MRSA/J strains, our aim being to identify a factor/structure specific to the strongly invasive SI1 pathotype. We also investigated bacterial surface structures of CA‐MRSA/J.

Clinical information on the four CA‐MRSA/J strains (NN3, NN4, NN50 and SI1) and 26 other strains is summarized in Table S1 and Table S2.^11^ Briefly, NN3 and NN4 were isolated from skin infections (without BSIs), NN50 was isolated from abscesses in the erector spinae muscles (with BSIs), and SI1 was isolated from rapidly progressing, fatal multi‐organ abscesses (with BSIs) (Table S1). The 26 other strains included seven strains from SSTIs (including atopic dermatitis and cellulitis), three from invasive infections (including iliopsoas abscesses and hydrothorax), seven from pneumonia/sputum, two from diarrhea, one from a urinary tract infection, three from nasal colonization, and three from environmental sources (trains) (Table S2).

The PCR primers used for analyses of virulence genes, SCC*mec*IVl structures and drug/antiseptic resistance are listed in Table S3.^12^, ^13^, ^14^, ^15^, ^16^, ^17^ Genes analyzed included staphylococcal superantigen genes on *S. aureus* pathogenicity island 18 and immune evasion cluster genes on phage Sa3.^15^ Susceptibility testing was performed according to previously described procedures.^8^, ^19^ The 30 antimicrobial and related agents tested (Table S4) included three β‐lactams, seven aminoglycosides, two macrolides/lincosamides, two glycopeptides, linezolid, daptomycin, levofloxacin, rifampicin, trimethoprim, sulfamethoxazole, tetracycline, fosfomycin, mupirocin, fusidic acid; and six antiseptics (including benzalkonium chloride) and related agents (ethidium bromide). Plasmids were transferred as described previously.^8^

The bacterial genomes from SI1 and NN3 were analyzed in 2016 and 2018, respectively, using the PacBio RS II system. The SCC*mec*IVl sequence of NN50 5, 6 was revised in this study. Previous NN50 sequence data, obtained by pyrosequencing genome sequencing technology with gap‐filling by PCR and sequencing, suggested three blocks of 86‐aa repeats for the *spj* gene;^5^ however, the present sequence analysis involving PCR and sequencing revealed five blocks of 86‐aa repeats for the NN50 *spj* gene (as described below). The aa repeat region of *spj* from NN4 was also PCR‐amplified and sequenced. The complete SI1 plasmid (pWSI1) sequence was determined in 2016. The complete SCC*mec*IVl sequences of SI1, NN3, and NN50 were deposited in GenBank under accession numbers LC425379, LC425378, and AB633329, respectively. For the *spj* sequence from NN4 and the complete pWSI1 sequence, GenBank accession numbers are LC440394 and LC383633. Antibodies against the Spj 11/22‐aa repeats (NN50), STEESTKEEQPSAEEVGKEAQS (N** → **C), were made in rabbits; the antibody‐bound cells having been detected by FITC‐labeled second antibody. A HEp‐2 cell infection assay was performed using SEM and TEM, as described previously.^8^ Data were analyzed statistically with Fisher's exact test. *P* < 0.05 was considered to denote significance.

NN3, NN4, NN50 and SI1 are all genotype ST8/SCC*mec*IVl/*agr*1/coagulase type III members with *spa* types 606/t1767, or 605/t12625 (for NN3) (Table S1). Divergence was noted in the immune invasion cluster genes and transposons (Table S1), and in plasmids (Table S1, Fig. S1).^20^ For example, NN4 and SI1 (but not NN3 and NN50) were found to carry a 32 kb plasmid (p32kb), which carries a virulence gene *edin* (or *ednA*) and an antiseptic resistance gene *qacB*. The drug resistance of the strains are summarized in Table S4; for example, NN3, NN4 and SI1 (but not NN50) were found to be resistant to bleomycin (due to *bleO*) and neomycin (due to *aadD*), whereas NN4 and SI1 (but not NN3 and NN50) exhibited resistance to benzalkonium chloride, acriflavin and ethidium bromide (due to *qacB*). They were found to be susceptible to the generally recommended anti‐MRSA agents.

The entire SCC*mec*IVl structures of NN3, NN50 and SI1 are shown in Figure 1. The *spj* gene in the joining 1 region was found to show size variation because of the variable 22‐ and 86‐aa repeat regions (Figure 1A). The 22‐aa repeat regions in NN3, NN4, NN50 and SI1 are shown in Figure 2 (A [left side], B [upper part], C, D). They consist of two 11‐aa basic units (α and β) and αβ_n_ and end with α and truncated β_1‐5_, α∆β (Figure 2C and D). One additional 11‐aa basic unit (γ) is present in NN3 and NN4 at the second repeat position, albeit alone (i.e., unconnected to α or β) (Figure 2C and D). SI1 lacks αβ repeats and retains only the last α∆β (Figure 2D). All basic units (α, β and γ) were found to start with an N terminal serine, whereas β and γ end with a C terminal serine. In contrast, α, which is always present as αβ, ends with proline (Figure 2C). Therefore, αβ and γ, which are defined herein as an S(X)_n_S motif, act as a repeating unit for constructing the 11/22‐aa repeat. On the basis of its 11‐aa components, the previous 22‐aa repeat is described as an 11/22‐aa repeat in this study.

**Figure 1:**
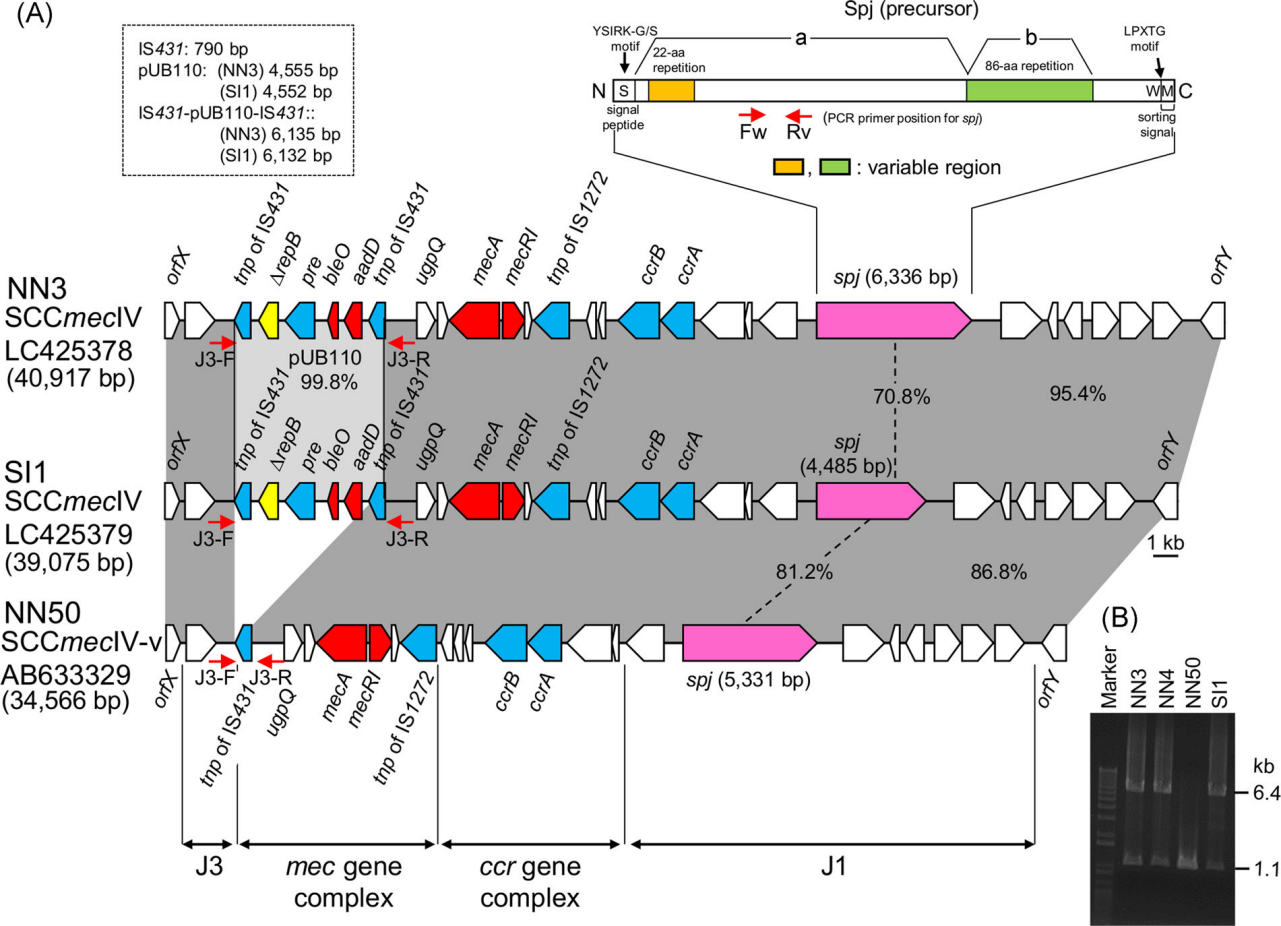
SCC*mec*IVl structures of ST8 CA‐MRSA/J NN3, SI1 and NN50 strains. (A), Homologous regions are shaded in each comparison. PCR primers Fw and Rv were used to detect the *spj* gene on SCC*mec*IVl.^5^, ^6^ The structure of the large CWA protein (Spj), encoded by the *spj* gene, has been drawn, above the *spj* gene, according to previously reported information.^5^ S, signal peptide; A, target/ligand‐binding; B, “stalk”; W, wall‐anchoring and wall‐spanning (LPXTG motif); M, membrane‐spanning. The two‐aa repeat domains in the A and B regions of Spj have been described previously.^5^ In this study, two variable regions (22‐ and 86‐aa repeats) were identified, and on the basis of the 11‐aa components in the 22‐aa repeats, the 22‐aa repeat was identified as an 11/22‐aa repeat. The IS*431*‐flanking pUB110 (IS*431*‐pUB110‐IS*431*) region on SCC*mec*IVl is also shown. PCR primers J3‐F and J3‐R were designed to detect the IS*431*‐flanking pUB110 region. *repB* from pUB110 was found to have a premature stop codon (mutation T793A) that results in a truncated product (∆RepB). SCC*mec*IVl of NN50 lacks IS*431*‐pUB110, retaining only IS*431*; the SCC*mec*IVl variant of NN50 is named SCC*mec*IVl‐v. (B), PCR results (using primers J3‐F and J3‐R) for NN3, NN4, NN50 and SI1 are shown. NN50 was found to lack a 6.4‐kb band (which indicated the presence of IS*431*‐flanking pUB110). NN3, NN4 and SI1 were found to produce a faint 1.1‐kb band, corresponding to that of SCC*mec*IVl‐v, suggesting the presence of pUB110‐deleted cells at low frequencies ( < 10^‐4^). [Color figure can be viewed at wileyonlinelibrary.com]

**Figure 2:**
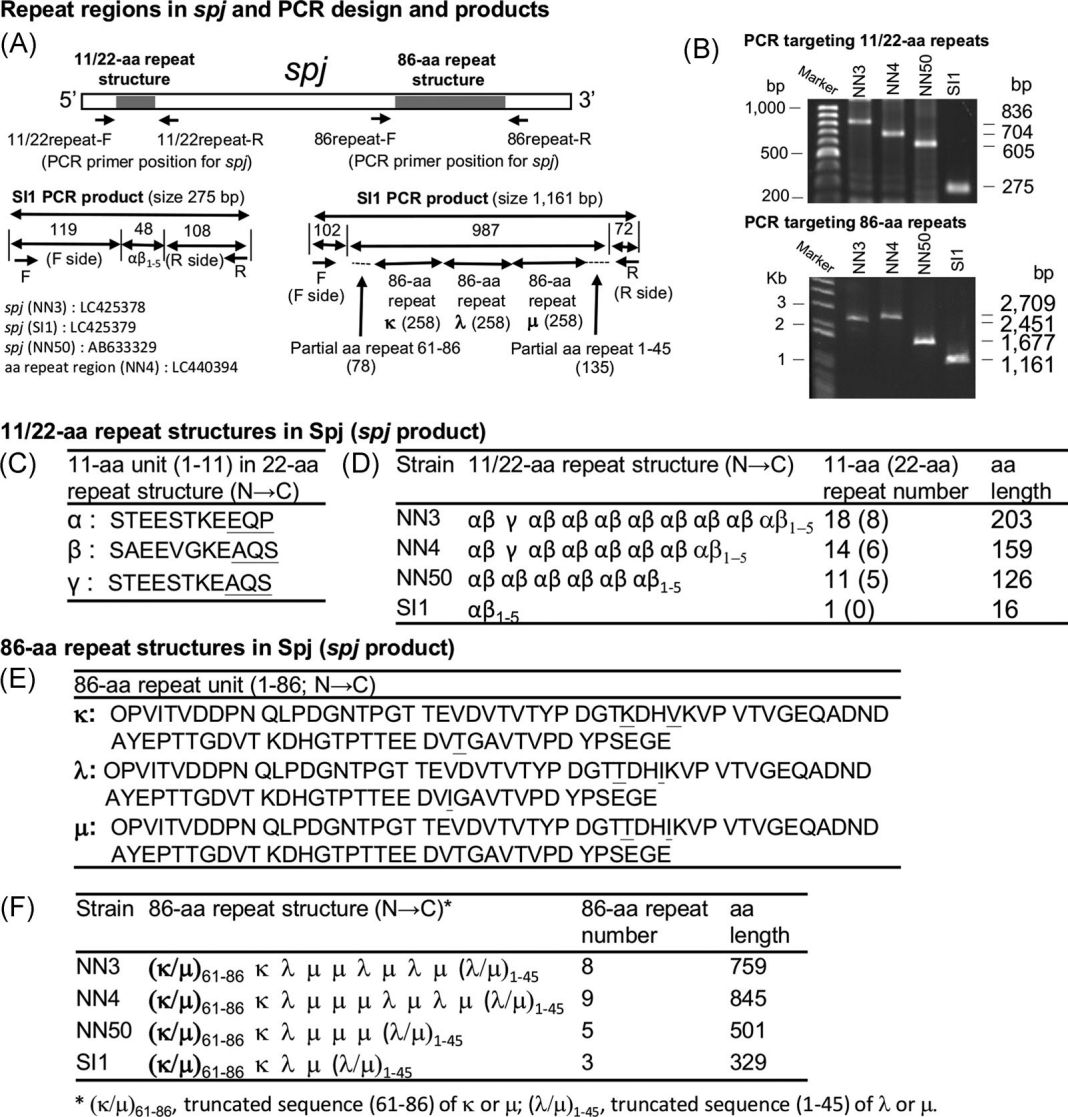
Structures of the 22‐ and 86‐aa repeat regions in the *spj* gene from CA‐MRSA/J strains NN3, NN4, NN50 and SI1. (A), The locations of the PCR primers (11/22repeat‐F and 11/22repeat‐R) and (86repeat‐F and 86repeat‐R) are shown. (B), Upper panel, PCR with primers 11/22repeat‐F and 11/22repeat‐R; lower panel, PCR with primers 86repeat‐F and 86repeat‐R. (C) and (D), The 11‐aa basic units (α, β,γ) comprising the 22‐aa repeat domain are summarized. The PCR target of SI1 (with primers 11/22repeat‐F and 11/22repeat‐R) is α∆β, and the predicted PCR product size is 275 bp, as shown in (A, left lower), (B, upper panel) and (D). (E) and (F), The 86‐aa basic units (κ, λ, μ) comprising the 86‐aa repeat domain are summarized. The PCR target of SI1 (with primers 86repeat‐F and 86repeat‐R) is ∆κ/μ‐κ‐λ‐μ‐∆λ/μ, containing three copies of the 86‐aa unit (κ, λ, μ), and the predicted PCR product size is 1161‐bp, as shown in (A, right lower), (B, lower panel) and (F). The targets (and PCR product size) for *spj* with two 86‐aa repeat units, one 86‐aa repeat unit, or lacking an 86‐aa repeat unit are, respectively, ∆κ/μ‐κ‐λ‐∆λ/μ (903 bp), ∆κ/μ‐κ‐∆λ/μ (645 bp) and ∆κ/μ‐∆λ/μ (387 bp)

In PCR with primers 11/22repeat‐F and 11/22repeat‐R, the αβ repeat‐lacking SI1 generated the smallest (275‐bp) product (Figure 2A [left side], B [upper part], and D). The same PCR for NN50, NN4 and NN3 generated larger products of 605, 704 and 836 bp, respectively (Figure 2B [upper part] and D). Another set of PCR primers, 11/22repeat‐F3 and 11/22repeat‐R3, essentially yielded similar results (Fig. S2A and B); in this case, the αβ repeat‐lacking SI1 generated the smallest (367‐bp) product, with larger products for NN50, NN4 and NN3 (697, 796, and 928 bp, respectively). The 11/22‐aa repeat structures are predicted to be strongly hydrophilic (Fig. S3). In each 11‐aa basic unit, glutamic acid accounts for 36.4% (4/11) for α and 27.3% (3/11) for β and γ (Figure 2C).

Data on a total of 30 CA‐MRSA/J strains are summarized in Table 1. The 11/22‐aa repeat structures were classified into three major groups (α, β, γ; α, β; and no repeat), with a total of eleven types. Of these, (αβ αβ αβ αβ αβ , αβ_5_) was found to be the most prevalent, accounting for 30.0% (9/30); (αβ γ αβ αβ αβ αβ αβ αβ αβ; ) was the second‐most prevalent (16.7%, 5/30). The α,β,γ group mainly involved isolates from SSTIs (38.5%, 5/13). Invasive strains had shorter αβ repeats (αβ_5_ or less). No repeat case included only SI1. Two prevalent types, (αβ γ αβ αβ αβ αβ αβ αβ; and (αβ_5_), involved isolates from public transport (trains). Two types in the α,β group involved isolates from diarrhea; these were related to isolates from retail meats.^11^

**Table 1:** The 11/22‐amino acid repeat structures in the *spj* gene from 30 CA‐MRSA/J strains isolated from various sources

Structure (type) of the 11/22‐aa repeat domain^a^ (N → C)	CA‐MRSA/J from clinical sources (diseases)						CA‐MRSA/J from environments
	Skin and soft tissue infection^b^	Pneumonia/sputum^c^	Nasal colonization (carrier)	Diarrhe^d^	Urine^c^	Invasive infection	
(*n* = strain number in each type)	*n* = 9	*n* = 7	*n* = 3	*n* = 2	*n* = 1	*n* = 5	*n* = 3
α, β, γ group							
αβ γ αβ αβ αβ αβ αβ αβ αβ; (*n* = 5)	2	1	2	0	0	0	0
αβ αβ γ αβ αβ αβ αβ αβ αβ; (*n* = 1)	1	0	0	0	0	0	0
αβ γ αβ αβ αβ αβ αβ αβ; (*n* = 4)	1	0	0	0	1	0	2
αβ γ αβ αβ αβ αβ αβ (*n* = 3)	1	2	0	0	0	0	0
α, β group							
αβ αβ αβ αβ αβ αβ αβ; (*n* = 1)	0	0	0	1	0	0	0
αβ αβ αβ αβ αβ αβ (*n* = 1)	1	0	0	0	0	0	0
αβ αβ αβ αβ αβ (*n* = 9)	3	1	1	0	0	3^e^	1
αβ αβ αβ αβ (*n* = 3)	0	2	0	1	0	0	0
αβ αβ αβ (*n*= 1)	0	0	0	0	0	1^f^	0
αβ (*n* = 1)	0	1	0	0	0	0	0
No repeat group							
(*n* = 1)	0	0	0	0	0	1^g^	0

CA‐MRSA/J strains used are those from references 6, 7, 8, 9, 10 and include the four strains in Table S1.

^a^ The C‐terminal αβ_1‐5_ is omitted in each case; however, all stains (*spj* genes) had the C‐terminal αβ_1‐5_.

^b^ Diseases include bullous impetigo, atopic dermatitis, skin abscesses, eczema, and cellulitis.

^c^ Clinical courses are not defined (because of lack of information).

^d^ Isolates are related to those from retail meats.^11^

^e^ Cases include abscesses at erector spinae muscles and epidural region of spine and sepsis from NN50; iliopsoas abscesses and discitis with thrombocytopenia from NN55;^7^ and postsurgical infection from NN44.^6^

^f^ Case is hydrothorax from 3963.^6^

^g^ Case is a fatal infection from SI1.

The 86‐aa repeat structures from NN3, NN4, NN50, and SI1 strains are summarized in Figure 2 (A [right side], B [lower part], E, F). The aa repeat structure was longest in NN4, followed by NN3, NN50 and SI1 (Figure 2B [lower part] and F), and is denoted as (N → C) partial repeat sequence (61–86)‐(86‐aa unit)_n_‐partial repeat sequence (1–45), where the 86‐aa unit consists of κ, λ and μ, the starting array being κ‐λ‐μ in each strain (Figure 2E and F). The 86‐aa unit was rich in acidic aa residues (aspartic and glutamic acids), accounting for 20.9% (18/86), and proline, accounting for 11.6% (10/86) (Figure 2E).

Next, the adherence and invasion properties of NN3, NN4, NN50, and SI1 were analyzed in a HEp‐2 cell assay (Figure 3). With NN50, some bacteria were found to elongate the microvilli of HEp‐2 cells and adhered to the elongated microvilli as bacterial aggregates (microcolony), as shown in Figure 3A to C (arrow), whereas some other NN50 cells (arrowhead in Figure 3A) were found to be wrapped in elongated HEp‐2 cell membrane (arrowhead in Figure 3B) and to be invading the cytoplasm of HEp‐2 cells (arrowhead in in Figure 3C). In the case of SEM, MRSA cells which invaded the HEp‐2 cell cytoplasm are not detected (from the HEp‐2 cell surface side); therefore, the initial stage of invasion (membrane‐wrapped MRSA), which is detected on the HEp‐2 cell surface by SEM, was evaluated as MRSA invasion. The levels of adherence to the elongated microvilli of HEp‐2 cells were the greatest in NN3 or NN4, followed by NN50 and SI1, whereas the levels of invasion of HEp‐2 cells were greatest in SI1, followed by NN50 and NN3 or NN4 (*P < *0.02) (Figure 3D). NN3, NN4, NN50 and SI1 were found to have a unique bacterial surface layer (surface X structure) with a thickness of ca. 75 nm, as shown in Figure 3E–I (arrowhead). Antibodies against Spj (NN50) were found to bind specifically to adherent NN50 cells (Figure 3J and K).

**Figure 3:**
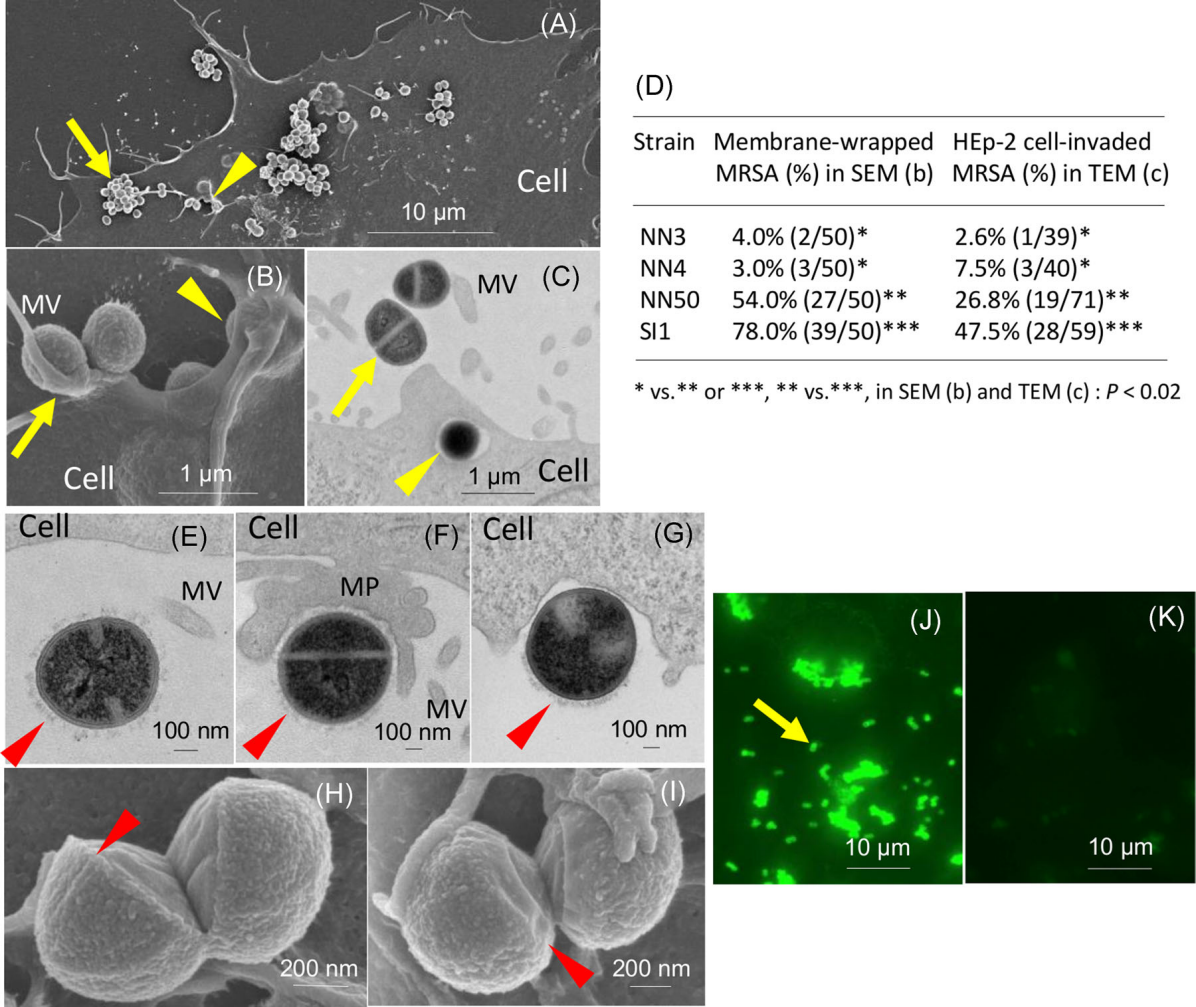
SEM, TEM and fluorescence micrographs showing adherence and invasion to HEp‐2 cells by CA‐MRSA/J strain NN50, and comparison of HEp‐2 cell invasion levels between CA‐MRSA/J strains NN3, NN4, NN50 and SI1. (A–C), NN50′ properties was examined. (A, B), SEM micrographs; (C), TEM micrographs. Arrows indicate bacterial adherence to the elongated microvilli (MV) above the HEp‐2 cell surface (as microcolony/bacterial aggregates). Arrowheads indicate bacterial invasion: (B), SEM showing that bacteria are wrapped by the elongated HEp‐2 cell membrane; (C), TEM showing that bacteria are located in the cytoplasm of HEp‐2 cells. (D), HEp‐2 cells were infected with NN3, NN4, NN50, or SI1. (E–G), TEM; arrowheads indicate a unique bacterial surface layer (surface X structure). (E), NN3 attaching MV; (F), NN4 interacting tightly with membranous cup‐like projection (MP, pedestal formation); and (G), SI1 invading the cytoplasm. (H, I), SEM; arrowheads indicate a unique bacterial surface layer. (H), NN50; (I), SI1. (J, K), Fluorescence (FITC) micrographs. (J), NN50 on HEp‐2 cells (arrow) treated with anti‐11/22‐aa repeats antibodies; (K), NN50 on HEp‐2 cells without antibody treatment. [Color figure can be viewed at wileyonlinelibrary.com]

Finally, the SCC*mec*IVl structures from NN3 and SI1 were found to have IS*431*‐flanking pUB110 (IS*431*‐ pUB110‐ IS*431*) in the J3 region (Figure 1A), a situation similar to that for SCC*mec*II from HA‐MRSA (Fig. S4).^21^ PCR analysis with J3‐F and J3‐R primers revealed that SCC*mec*IVl from NN4 also carries IS*431*‐flanking pUB110 (Figure 1A and B). In contrast, SCC*mec*IVl from NN50 (named SCC*mec*IVl‐v) was found to lack IS*431*‐pUB110, retaining only IS*431* (Figure 1A and B); this was attributable to recombination between two directly oriented copies of IS*431* (Fig. S4). When 26 other CA‐MRSA/J strains were also examined, pUB110 carriage of 90.0% was found (27/30).

SI1 is susceptible to the recommended anti‐MRSA agents; however, disease caused by it progresses rapidly, leading to uncontrollable sepsis with unique pathologic features, such as pulmonary embolism with SI1 bacterial aggregates in the pulmonary blood vessels, endocarditis accompanied by thrombus formation, and multiple organ abscesses and failure.^8^ In contrast, NN3 and NN4 were isolated from bullous impetigo, a common localized blistering skin disease in children, although they (and other CA‐MRSA/J) were found to be negative for the genes encoding exfoliative toxin, a major cause of bullous impetigo,^22^ and collagen adhesin, a frequently‐found factor.^23^, ^24^

CWA proteins, which are covalently linked to peptidoglycan, play a role in bacterial adherence, invasion and immune evasion.^25^ In the present study, we demonstrated that the two aa‐repeat domains of Spj, a CWA protein, are highly variable and clearly reflect its clinical origins, in that those of SI1 are short whereas those of NN3 and NN4 are long. Of the two repeats, the 11/22‐aa repeats more clearly distinguish SI1 from NN3 or NN4. This phenomenon is parallel to CA‐MRSA/J's properties in an *in vitro* HEp‐2 cell assay (strongly invasive SI1 vs. microvilli‐adherent NN3 and NN4). Moreover, in the present study, the 11/22‐aa repeat structures were classified into three major groups with a total of eleven types, suggesting novel *spj* typing for CA‐MRSA/J infections and spread.

Rapid diagnosis is important for strongly invasive infections. In the present study, SI1 had the shortest 11/22‐aa repeat structure among the CA‐MRSA/J strains examined. On the basis of this fact, we have developed a PCR (targeting the short 11/22‐aa repeat region of *spj*) for rapid diagnosis of strongly invasive SI1.

It is possible that variable Spj is a strong adherence/invasion factor; however, additional experiments using ∆*spj* mutants of CA‐MRSA/J (such as SI1 and NN3 strains) are required for further consideration of the role of a variable Spj in virulence. CA‐MRSA/J has a unique bacterial surface layer (surface X). The molecular and genetic features of “surface X” are currently being investigated.

From an evolutionary viewpoint, it is noteworthy that the ST5/SCC*mec*II HA‐MRSA lineage,^3^, ^18^, ^26^ which is predominant in Japan, and CA‐MRSA/J share similar features, with IS*431*‐flanking pUB110 (*bleO*, *aadD*) and the staphylococcal superantigen gene cluster (*tst*, *sec*, *sell*), suggesting selective advantages. The addition of the variable *spj* gene and a unique bacterial surface layer (surface X) likely make CA‐MRSA/J successful in community settings.

## DISCLOSURE

The authors declare that they have no conflicts of interest.

## Supporting information

Supporting information
