# Supplementary material for: Structures of a highly variable cell‐wall anchored protein‐encoding the spj gene from ST8/SCCmecIVl community‐associated methicillin‐resistant Staphylococcus aureus (CA‐MRSA/J) isolated from 2003 onwards: An indicator of a strongly invasive pathotype
Source: Microbiol Immunol. 2019 May 23;63(5):186–93. doi: 10.1111/1348-0421.12684 (PMC6617794; doi:10.1111/1348-0421.12684)
Supplement: Supplementary file 1 — Supporting information [file MIM-63-186-s001.pdf]

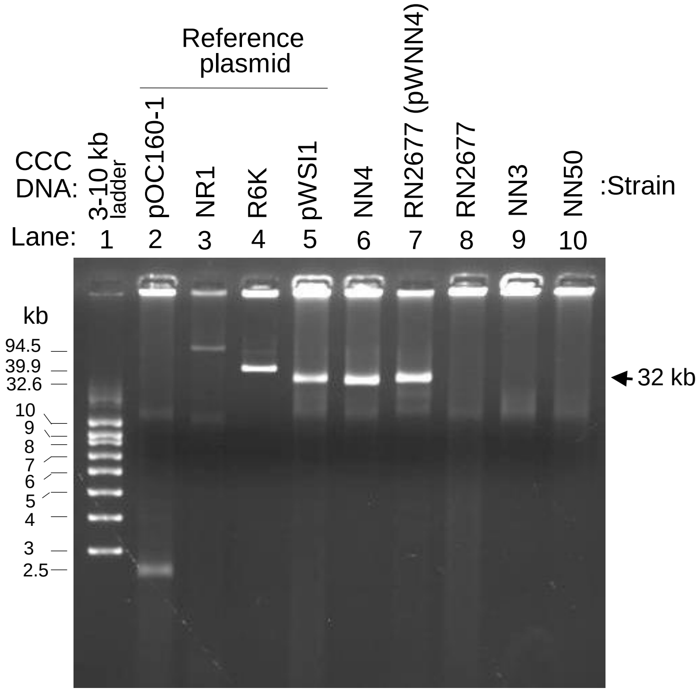

Fig. S1. Covalently closed circular (CCC) plasmid DNA analysis of CA-MRSA/J strains NN3, NN4, and NN50. NN4, which carries the 32-kb plasmid (pWNN4), was mixed with *S. aureus* RN2677 and transconjugants (RN2677 carrying pWNN4) were selected with ethidium bromide and novobiocin. The transfer frequency (transconjugants/donor) was 7.5×10^{-6}. The transfer protocol for the pWSI1 plasmid from SI1 was described previously (8). In this study, pWSI1 (32,573-bp) was used as a reference plasmid (size marker), together with the 2,476-bp pOC160-1 plasmid (8, 20) and size-known NR1 and R6K.

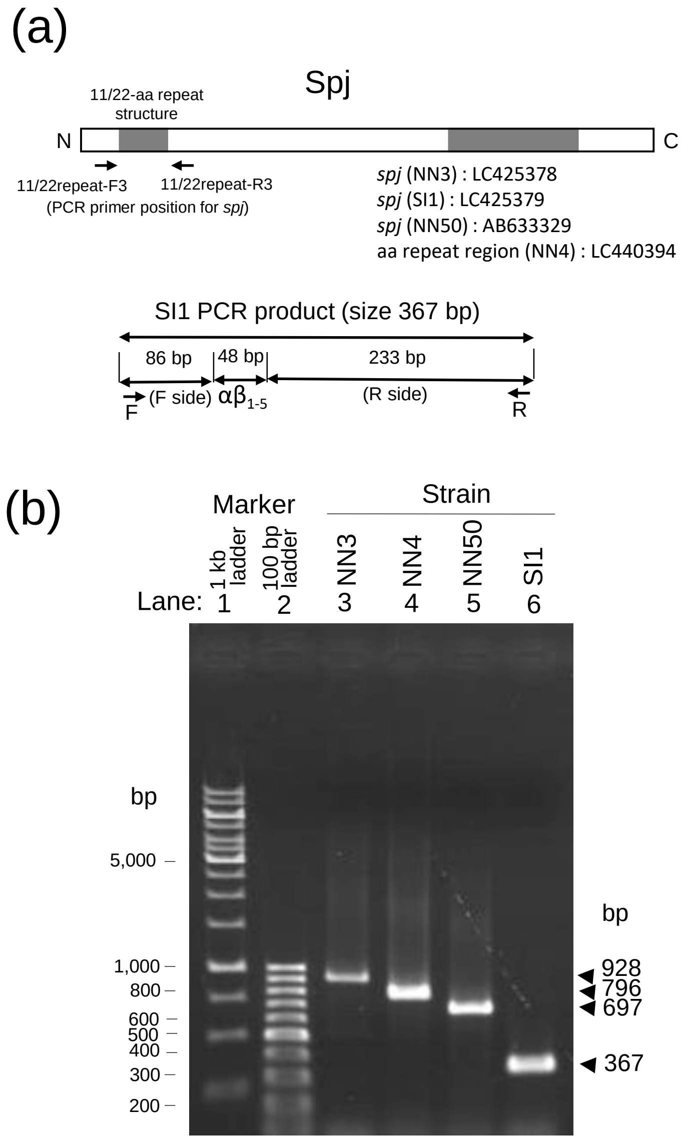

Fig. S2. PCR analysis of the 11/22-amino acid (aa) repeat region of the *spj* gene from CA-MRSA/J strains NN3, NN4, NN50, and SI1. In (a), the locations of the PCR primers (11/22repeat-F3 and 11/22repeat-R3) are shown. In (b), PCR with primers 11/22repeat-F3 and 11/22repeat-R3 was performed for CA-MRSA/J strains NN3, NN4, NN50, and SI1.

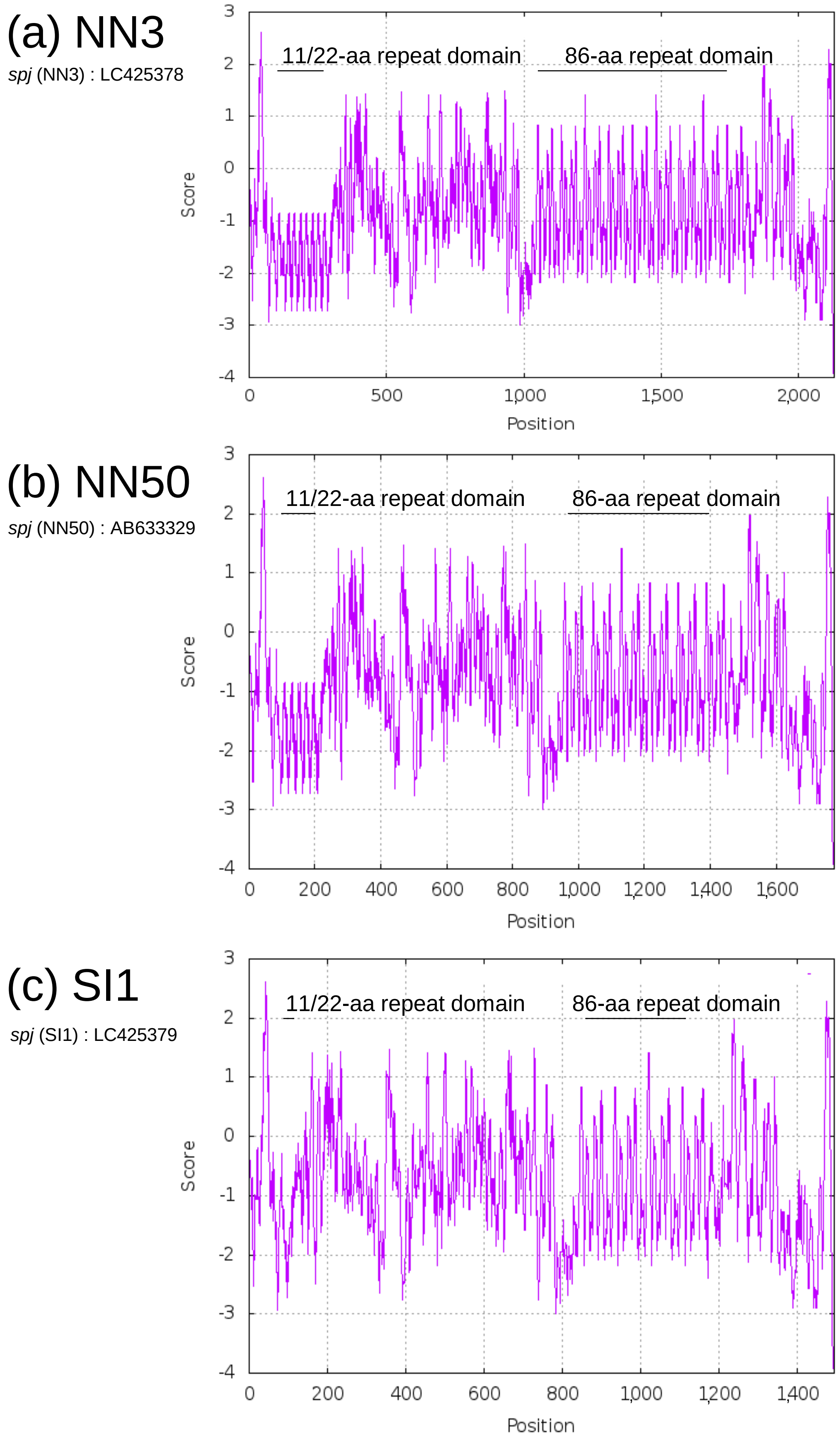

Fig. S3. Hydrophobicity patterns of the *spj* products (Spj precursors) for CA-MRSA/J strains NN3 (a), NN50 (b), and SI1 (c). Horizontal lines in each figure show the position of the 11/22-amino acid (aa) and 86-aa repeat domains.

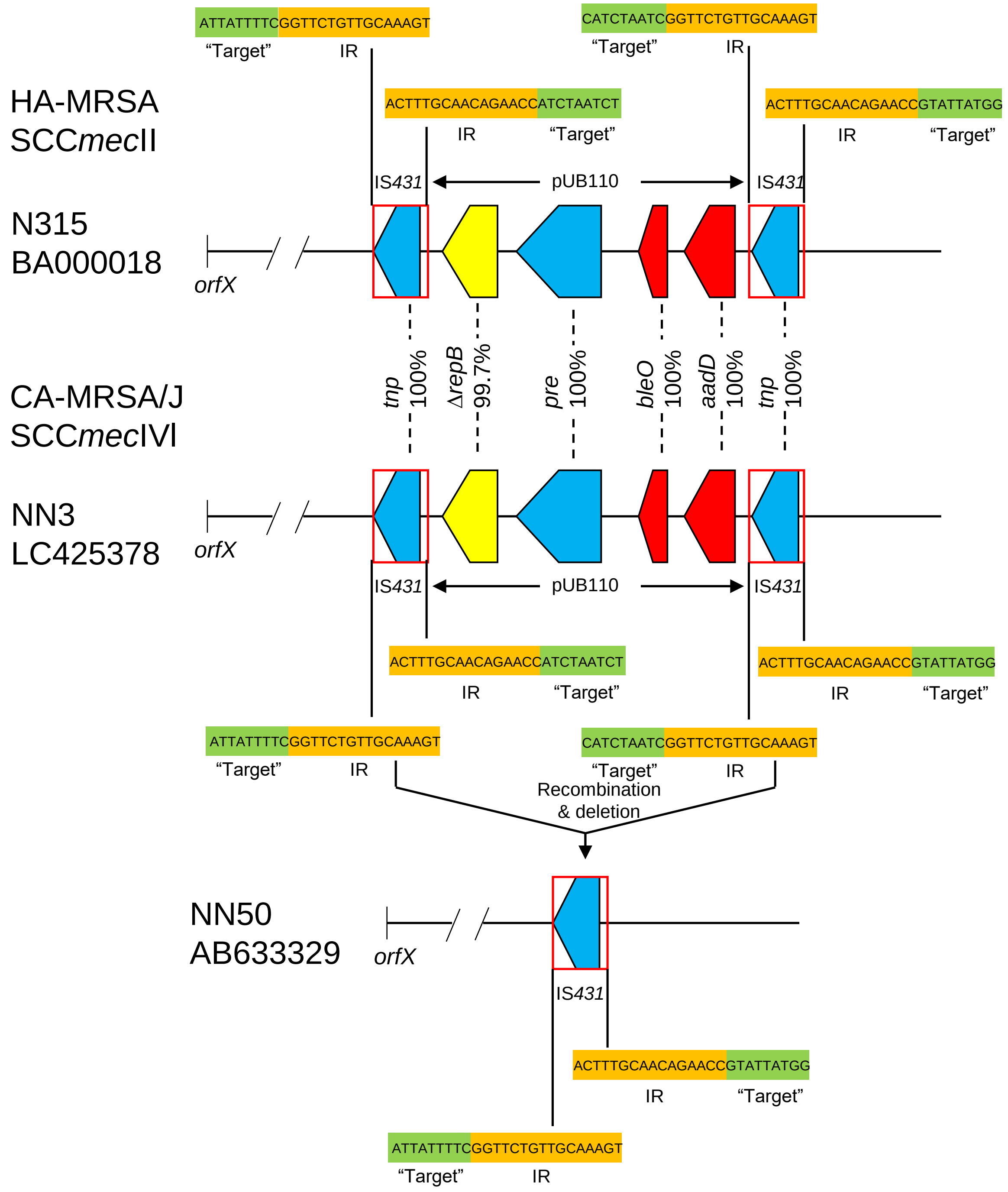

Fig. S4. Structures of the IS*431*-flanked pUB110 region from CA-MRSA/J strains NN3 and NN50, in comparison with the ST5/SCC*mec*II HA-MRSA strain N315. Homologies (%) are at the nucleotide levels. IS*431* (also known as IS*257*) contains the perfect terminal 16-bp inverted repeats (IRs), and may be flanked by a directly oriented 8-bp target sequence (21). However, comparing the IS*431* copies between NN3 and N315 allowed us to assign the 9-bp "target" sequence. The 9-bp "target" sequence was not duplicated. The structure of the IS*431*-flanked pUB110 region from SI1 was the same as that from NN3.

Table S1. Relevant characteristics of ST8/SCC*mec*IVl CA-MRSA (CA-MRSA/J) strains NN3, NN4, NN50, and SI1

Isolation, genotype, plasmid, resistance	Strain NN3	Strain NN4	Strain NN50	Strain SI1
(Clinical information)				
Case year	2003	2003	2008	2012
Associated disease	Bullous impetigo	Bullous impetigo	Abscesses at erector spinae muscles and epidural region of spine, sepsis	Iliopsoas abscesses, septic pulmonary embolism, multiorgan abscesses
MRSA isolation from blood	-	-	+	+
Patient (age, sex)	1 Y, M	7 M, M	11 Y, M	64 Y, F
Outcome	Recovery	Recovery	Recovery	Death
Reference	10	10	6	8
(MRSA data)				
ST/*spa*/*agr*/coagulase type	8/605(t12625)/1/III	8/606(t1767)/1/III	8/606(t1767)/1/III	8/606(t1767)/1/III
SCC*mec* type	IVl	IVl	IVl –v (ΔpUB110) †	IVl
blaZ (Tn*552*)	+	+	+	+
tst/sec/selI (SaPI)	+	+	+	+
Immune evasion cluster (type)	*sak, scn* (E)	*sak, scn, sep* (G)	*sak, scn, sep* (G)	*sak, scn, chp* (B)
edin (*ednA*)	-	+	-	+
PFGE type‡	γ	α	β	γ
Plasmid (p32kb) §	-	+ (pWNN4)	-	+ (pWSI1)
Non–β-lactam resistance ¶ (gene)				
Genetic trait:				
pUB110 (SCC*mec*IVl)	BLE (*bleO*), KAN/TOB/NEO (*aadD*)	BLE (*bleO*), KAN/TOB/NEO (*aadD*)	-	BLE (*bleO*), KAN/TOB/NEO (*aadD*)
Tn*4001*	-	GEN/KAN/TOB (*aacA-aphD*)	GEN/KAN/TOB (*aacA-aphD*)	-
Tn*554*	-	-	-	ERY/CLI ind (*ermA*), SPE (*aad9/spc*)
p32kb	-	BEN/ACR/ETH (*qacB*)	-	BEN/ACR/ETH (*qacB*)

† SCC*mec*IVl-v, SCC*mec*IVl variant lacking pUB110.

‡ PFGE, pulsed-field gel electrophoresis. Data are from references 6 and 8.

§ p32kb, a transmissible 32-kb plasmid (carrying genes *qacB* and *edin*/*ednA*).

¶ BLE, bleomycin; KAN, kanamycin; TOB, tobramycin; NEO, neomycin; GEN, gentamicin; ERY, erythromycin; CLI, clindamycin, ind, inducible; SPE, spectinomycin; BEN, benzalkonium/benzethonium chloride; ACR, acriflavin; ETH, ethidium bromide.

Table S2. Summary of 30 CA-MRSA/J strains isolated from various sources and the 11/22-amino acid repeat structures in the *spj* gene

Structure (type) of the 11/22-aa repeat domain[†] (N→C) in the *spj* gene (11-aa/22-aa repeat number) n = strain number in each type	CA-MRSA/J from clinical sources (diseases)									CA-MRSA/J from environments		Reference
	Strain	Patient' age	Disease: Skin and soft tissue infection	Pneumonia/ sputum	Colonization	Intestinal tract infection	Urinary tract infection	Invasive infection	Outcome	Strain	Isolation	
α,β,γ group												
αβ γ αβ αβ αβ αβ αβ αβ αβ (17/8) n = 5	NN3	1 Y	Bullous impetigo						Recovery			6, 10
	TI-5	21 Y	Atopic dermatitiss						Recovery			6
	3457	15 Y		Necrotizing pneumonia					Recovery			6
	2F5	30 Y			Nares[‡]				-[§]			6
	2I5	31 Y			Nares[‡]				-[§]			6
αβ γ αβ αβ αβ αβ αβ αβ (15/7) n = 4	NN39	1 Y	Bullous impetigo						Recovery			6
	809	5 M					Urine		Recovery			6
										PT3	Train	9
										PT4	Train	9
αβ γ αβ αβ αβ αβ αβ (13/6) n = 3	NN4	7 M	Bullous impetigo						Recovery			6, 10
	571	1 M		Sputum[¶]					Recovery			6
	737	1 M		Sputum[¶]					Recovery			6
αβ αβ γ αβ αβ αβ αβ αβ αβ (17/8) n = 1	2F4	2 Y	Abscess						Recovery			6
α,β group												
αβ αβ αβ αβ αβ αβ αβ (14/7) n = 1	S2	11 Y				Diarrhea[††]			Recovery			6
αβ αβ αβ αβ αβ αβ (12/6) n = 1	NN19	8 Y	Bullous impetigo						Recovery			6
αβ αβ αβ αβ αβ (10/5) n = 9	NN50	11 Y						Abscesses at erector spinae muscles and epidural region of spine, sepsis	Recovery			6
	NN55	62 Y						Iliopsoas abscess and discitis with thrombocytopenia	Recovery			7
	NN44	15 Y						Postsurgical infection	Recovery			6
	5184	51 Y	Cellulitis						Recovery			6
	5320	86 Y	Cellulitis						Recovery			6
	6400	3 Y		Sputum[¶]					Recovery			6
	2A3	7 Y	Eczema						Recovery			6
	9946	70 Y			Nares[‡]				-[§]			6
										PT5	Train	9
αβ αβ αβ αβ (8/4) n = 3	5135	3 Y		Pneumonia					Recovery			6
	3826	3 M		Sputum[¶]					Recovery			6
	S3	16 Y				Diarrhea[††]			Recovery			6
αβ αβ αβ (6/3) n = 1	3963	84 Y						Hydrothorax	Recovery			6
αβ (2/1) n = 1	658	92 Y		Sputum[¶]					Recovery			6
No repeat group												
n = 1	SI1	64 Y						Iliopsoas abscesses, septic pulmonary embolism, multiorgan abscesses	Death			8

[†] The C-terminal $\alpha\beta_{1-5}$ is omitted in each case, although all the stains (*spj* genes) had the C-terminal $\alpha\beta_{1-5}$.
[‡] Carrier.
[§] Not followed up.
[¶] Not defined (due to lack of information).
[††] Related to CA-MRSA/J isolates from retail meats (11).

Table S3. Primers used for the PCR assaying of virulence genes, the SCC*mec*IVl structure, and bacterial resistance genes

Primer (F/R set)	Primer sequence (5'→3')	Product size (bp)	Target	Reference
Virulence gene				
tst-GTSSTR-1	ACCCCTGTTCCCTTATCATC	326	*tst* (SaPI)	12
tst-GTSSTR-2	TTTTCAGTATTTGTAACGCC			
sec-1	GTAAAGTTACAGGTGGCAAAACTTG	296	*sec* (SaPI)	13
sec-2	CATATCATACCAAAAAGTATTGCCGT			
sel-F	CACCAGAATCACACCGCTTA	240	*sell* (SaPI)	14
sel-R	CTGTTTGATGCTTGCCATTG			
sak-1	AAGGCGATGACGCGAGTTAT	223	*sak* (IEC)	15
sak-2	GCGCTTGGATCTAATTCAAC			
scn-1W	GAAAATCTATACTTGCGGGA	253	*scn* (IEC)	This study
scn-2W	GACTTAAGAGCATACATTGC			
chp-1	TTTACTTTTGAACCGTTTCCTAC	366	*chp* (IEC)	15
chp-2W	TTAGTATGCATATTCATTAGTTTTTC			This study
sep-1	AATCATAACCAACCGAATCA	500	*sep* (IEC)	15
sep-2	TCATAATGGAAGTGCTATAA			
EDIN-1	GAAGTATCTAATACTTCTTTAGCAGC	620	*edin/ednA* (p32kb)	13
EDIN-2	TCATTTGACAATTCTACACTTCCAAC			
SCC*mec*IVl structue				
J3-F	AGTGAAATCAAACGGGAG	1.1k or 6.4k	pUB110 region	This study
J3-R	TCCTACTGCTCCTGAACC			
Fw	TGACCTCCAAGTAACAAAAG	485	*spj*	5
Rv	TCATCGTTACGTTACTTGGT			
11/22repeat-F	ATGAAGCGAAAGCAGACG	275 to 836	11/22-aa repeat region	This study
11/22repeat-R	GCGAATGACTTCTTCAGC			
11/22repeat-F3	CAGAGCAGGTATCTTCAG	367 to 928	11/22-aa repeat region	This study
11/22repeat-R3	TTAGGTGAAGCAGCTCTC			
86repeat-F	GTACAAGACGATGACGAG	1,161 to	86-aa repeat region	This study
86repeat-R	CACTTTGACTGTTTCTCCTG	2,451		
Bacterial resistance				
blaZ-r	AGTCTTGCCGAAAGCAGC	2547	*blaZ* (Tn*552*)	This study
blaI-r	GTCTTGTGATTAATGTTCTAATCG			
mecA354	AGATTGGGATCATAGCGT	717	*mecA* (SCC*mec*)	8
mecA1091	GGTGTGCTTACAAGTGCTA			
bleO-f	CATTGCCAGTCGGGGAT	341	*bleO* (pUB110)	This study
bleO-r	GTCGGGATCAATTACTGC			
aadD-f	AGAGTTCAGCCATGAATGG	551	*aadD* (pUB110)	8
aadD-r	CCACTATATATCCGTGTCG			
aacA-aphDF	ATACAGAGCCTTGGGAAG	499	*aacA-aphD* (Tn*4001*)	8
aacA-aphDR	GCCACACTATCATAACCAC			
ermA-f	TCTAAAAAGCATGTAAAAGAA	645	*ermA*(Tn*554*)	16
ermA-r	CTTCGATAGTTTATTAATATTAGT			
aadD-f	AGAGTTCAGCCATGAATGG	551	*aad9/spc* (Tn*554*)	This study
aadD-r	CCACTATATATCCGTGTCG			
qacA/B-f	GCAGAAAGTGCAGAGTTCG	361	*qacB* (p32kb)	17
qacA/B-r	CCAGTCCAATCATGCCTG			

Table S4. Minimum inhibitory concentrations (MICs) on antimicrobial agent testing of CA-MRSA/J strains (NN3, NN4, NN50, SI1) and *S. aureus* RN2677, which carries plasmid p32kb (pWNN4 from strain NN4 or pWSI1 from strain SI1)

Antimicrobial and other agents	MICs (μg/ml) for strains: NN3	NN4	NN50	SI1	RN2677 (pWNN4)	RN2677 (pWSI1)	RN2677
Penicillins							
Ampicillin	**16**†	**8**†	**16**†	**8**†	0.06	0.06	0.06
Oxacillin	**64**‡	**32**‡	**32**‡	**32**‡	0.13	0.13	0.13
Imipenem	4	0.5	0.25	0.5			
Macrolides/lyncosamides							
Erythromycin	0.5	0.5	0.5	**≥256** §	-¶	0.5	0.5
Clindamycin	0.13	0.13	0.13	0.13	-¶	0.13	0.13
Clindamycin + Erythromycin (1 μg/ml)	≤0.004	≤0.004	≤0.004	**16** (ind) §	-¶	≤0.004	≤0.004
Aminoglycosides							
Gentamicin	0.5	**32**††	**64**††	0.5	0.25	0.25	0.25
Arbekacin	1	1	1	0.5			
Kanamycin	**128**‡‡	**≥256**††, ‡‡	**≥256**††	**128**‡‡	2	2	2
Tobramycin	**≥256**‡‡	**≥256**††, ‡‡	**16**††	**≥256**‡‡	0.25	0.25	0.25
Neomycin	**128**‡‡	**64**‡‡	1	**128**‡‡	0.5	0.5	0.5
Spectinomycin	64	64	64	**≥256** § §	64	64	64
Streptomycin	8	8	8	8	-¶	-¶	-¶
TMP-SMX							
Trimethoprim	0.5	1	1	1	-¶	-¶	-¶
Sulfamethoxazole	8	16	8	16	-¶	-¶	-¶
Glycopeptides							
Vancomycin	1	1	1	1	-¶	-¶	-¶
Teicoplanin	2	1	1	1	-¶	-¶	-¶
Linezolid	2	2	2	2	-¶	-¶	-¶
Daptomycin	1	1	1	1			
Tetracycline	0.25	0.25	0.25	0.25	-¶	-¶	-¶
Levofloxacin	0.13	0.13	0.13	0.13	-¶	-¶	-¶
Rifampicin	0.03	0.03	0.03	0.03	-¶	-¶	-¶
Fosfomycin	2	2	2	2	-¶	-¶	-¶
Fusidic acid	0.13	0.13	0.13	0.13	-¶	-¶	-¶
Mupirocin	0.06	0.06	0.06	0.06	-¶	-¶	-¶
Others							
Bleomycin	**≥256**¶¶	**≥256**¶¶	32	**≥256**¶¶	1	2	4
Chlorhexidine gluconate	4	4	2	4	2	2	2
Benzalkonium chloride	2	**8**†††	2	**8**†††	**4**†††	**4**†††	2
Benzethonium chloride	2	**8**†††	2	**8**†††	**8**†††	**8**†††	2
Acriflavin	32	**256**†††	32	**256**†††	**256**†††	**256**†††	32
Ethidium bromide	8	**128**†††	8	**128**†††	**128**†††	**128**†††	8

Resistance, due to the presence of each resistance gene, or MIC values higher than those of the control strains (ATCC29213 or RN2677) is shown in boldface. † Resistance encoded by *blaZ* (Tn*552*). ‡ Resistance encoded by *mecA* (SCC*mec*IV). § Resistance encoded by *ermA* (Tn*554*). ¶ Data not examined. †† Resistance encoded by *aacA-aphD* (Tn*4001*). ‡‡ Resistance encoded by *aadD* (pUB110). § § Resistance encoded by *aad9*/*spc* (Tn*554*). ¶¶ Resistance encoded by *bleO* (pUB110). ††† Resistance encoded by *qacB* (p32kb).
